# *catena*-Poly[[aqua­(2-iodo­benzoato-κ*O*)cobalt(II)]-μ-aqua-μ-2-iodo­benzoato-κ^2^ *O*:*O*′]

**DOI:** 10.1107/S1600536812015115

**Published:** 2012-04-18

**Authors:** Ömür Aydın, Nagihan Çaylak Delibaş, Hacali Necefoğlu, Tuncer Hökelek

**Affiliations:** a Department of Chemistry, Kafkas University, 36100 Kars, Turkey; b Department of Physics, Sakarya University, 54187 Esentepe, Sakarya, Turkey; c Department of Physics, Hacettepe University, 06800 Beytepe, Ankara, Turkey

## Abstract

The asymmetric unit of the polymeric title compound, [Co(C_7_H_4_IO_2_)_2_(H_2_O)_2_]_*n*_, contains one Co^II^ cation, two iodo­benzoate anions and two water mol­ecules. One iodo­benzoate anion and one water mol­ecule bridge adjacent Co cations, forming a polymeric chain running along the *a* axis, while the other iodo­benzoate anion and water mol­ecule coordinate in a monodentate manner to the Co^II^ cation, completing the slightly distorted octa­hedral geometry. In the two independent anionic ligands, the carboxyl­ate groups are twisted away from the attached benzene rings by 51.38 (18) and 39.89 (11)°, and the two benzene rings are nearly perpendicular to each other with a dihedral angle of 86.09 (10)°. Intra­molecular O—H⋯O hydrogen bonds between coordinating water mol­ecules and adjacent carboxyl­ate O atoms help to stabilize the mol­ecular structure. In the crystal, weak C—H⋯O hydrogen bonds link the polymeric chains into a three-dimentional supra­molecular network.

## Related literature

For niacin, see: Krishnamachari (1974 ▶) and for information on the nicotinic acid derivative *N*,*N*-diethyl­nicotinamide, see: Bigoli *et al.* (1972 ▶). For related structures, see: Hökelek *et al.* (2009 ▶, 2010**a* ▶,b* ▶, 2011 ▶); Necefoğlu *et al.* (2011 ▶); Zaman *et al.* (2012 ▶). For bond-length data, see: Allen *et al.* (1987 ▶).
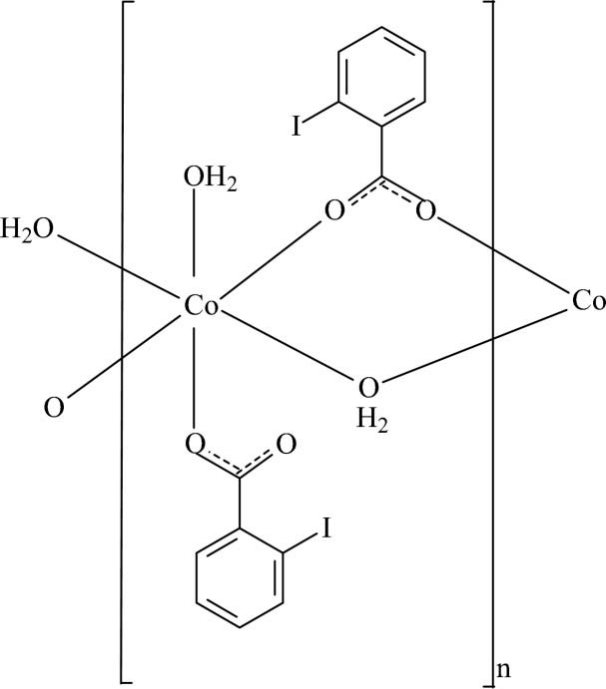


## Experimental

### 

#### Crystal data

[Co(C_7_H_4_IO_2_)_2_(H_2_O)_2_]
*M* *_r_* = 588.97
Orthorhombic, 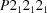

*a* = 7.5051 (3) Å
*b* = 10.5639 (4) Å
*c* = 21.6723 (9) Å
*V* = 1718.25 (12) Å^3^
*Z* = 4
Mo *K*α radiation
μ = 4.62 mm^−1^
*T* = 100 K
0.26 × 0.23 × 0.17 mm

#### Data collection

Bruker Kappa APEXII CCD area-detector diffractometer
Absorption correction: multi-scan (*SADABS*; Bruker, 2005 ▶) *T*_min_ = 0.313, *T*_max_ = 0.456
30167 measured reflections
4313 independent reflections
4300 reflections with *I* > 2σ(*I*)
*R*_int_ = 0.037

#### Refinement

*R*[*F* ^2^ > 2σ(*F* ^2^)] = 0.020
*wR*(*F* ^2^) = 0.051
*S* = 1.20
4313 reflections
224 parameters
8 restraints
H atoms treated by a mixture of independent and constrained refinement
Δρ_max_ = 0.60 e Å^−3^
Δρ_min_ = −1.03 e Å^−3^
Absolute structure: Flack (1983 ▶), 1835 Friedel pairs
Flack parameter: 0.016 (19)

### 

Data collection: *APEX2* (Bruker, 2007 ▶); cell refinement: *SAINT* (Bruker, 2007 ▶); data reduction: *SAINT*; program(s) used to solve structure: *SHELXS97* (Sheldrick, 2008 ▶); program(s) used to refine structure: *SHELXL97* (Sheldrick, 2008 ▶); molecular graphics: *ORTEP-3 for Windows* (Farrugia, 1997 ▶); software used to prepare material for publication: *WinGX* (Farrugia, 1999 ▶) and *PLATON* (Spek, 2009 ▶).

## Supplementary Material

Crystal structure: contains datablock(s) I, global. DOI: 10.1107/S1600536812015115/xu5504sup1.cif

Structure factors: contains datablock(s) I. DOI: 10.1107/S1600536812015115/xu5504Isup2.hkl

Additional supplementary materials:  crystallographic information; 3D view; checkCIF report

## Figures and Tables

**Table 1:** Selected bond lengths (Å)

Co1—O2	2.118 (2)
Co1—O3^i^	2.021 (2)
Co1—O4	2.016 (2)
Co1—O5	2.124 (2)
Co1—O5^i^	2.151 (2)
Co1—O6	2.110 (2)

Symmetry code: (i) 
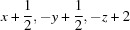
.

**Table 2:** Hydrogen-bond geometry (Å, °)

*D*—H⋯*A*	*D*—H	H⋯*A*	*D*⋯*A*	*D*—H⋯*A*
O5—H51⋯O1^ii^	0.86 (3)	1.64 (3)	2.486 (3)	167 (5)
O6—H61⋯O2^i^	0.86 (2)	1.92 (2)	2.742 (3)	161 (4)
C4—H4⋯O1^iii^	0.95	2.56	3.364 (4)	142
C13—H13⋯O4^iv^	0.95	2.58	3.495 (4)	162

Symmetry codes: (i) 
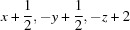
; (ii) 
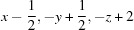
; (iii) 
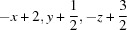
; (iv) 
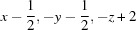
.

**Fig. 1.**
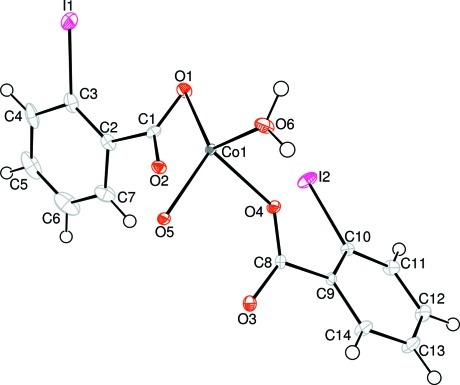
The asymmetric unit of the title molecule with the atom-numbering scheme. Displacement ellipsoids are drawn at the 50% probability level.

**Fig. 2.**
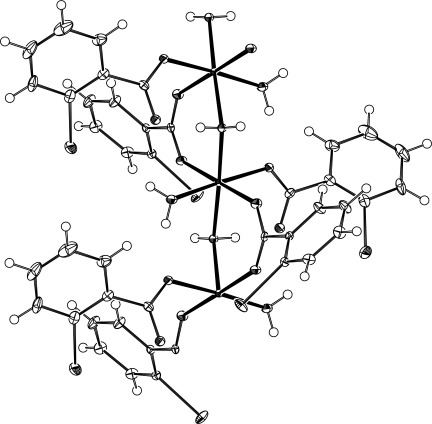
Part of the polymeric chain of the title compound.

## supplementary crystallographic information

### Comment

As a part of our ongoing investigations of transition metal complexes of nicotinamide (NA), one form of niacin (Krishnamachari, 1974), and/or the nicotinic acid derivative *N*,*N*-diethylnicotinamide (DENA), an important respiratory stimulant (Bigoli *et al.*, 1972), the title compound was synthesized and its crystal structure is reported herein.

The asymmetric unit of the title compound, (I), contains one Co^II^ ion, two 2-iodobenzoate (IB) ligands and two water molecules (Fig. 1). In the crystal of the title compound, each Co^II^ ion is coordinated by one IB ligand, one water molecule, two symmetry related IB ligands and two symmetry related water molecules, while the symmetry related Co(II) ions are bridged by the two O atoms of the symmetry related IB ligands and the two O atoms of the two symmetry related water molecules forming a polymeric chain. The coordination around the Co(II) ion is a slightly distorted octahedral (Fig. 2).

The crystal structures of some benzoate containing polymeric complexes of Mn^II^, Zn^II^ and Pb^II^ ions, [Mn_2_(C_8_H_7_O_2_)_4_(C_10_H_14_N_2_O)_2_(H_2_O)]_n_ (Hökelek *et al.*, 2010*a*), [Mn(C_7_H_4_FO_2_)_2_(H_2_O)_2_]_n_ (Necefoğlu *et al.*, 2011), [Zn(C_8_H_5_O_3_)_2_(C_6_H_6_N_2_O)]_n_ (Hökelek *et al.*, 2009), [Pb(C_8_H_7_O_2_)_2_(C_6_H_6_N_2_O)]_n_ (Hökelek *et al.*, 2010*b*), {[Pb(C_9_H_9_O_2_)_2_(C_6_H_6_N_2_O)].H_2_O}_n_ (Hökelek *et al.*, 2011), {[Pb(C_7_H_5_O_3_)_2_(C_6_H_6_N_2_O)].H_2_O}_n_ (Zaman *et al.*, 2012) have also been reported.

In the title compound, the four O atoms (O2, O3', O4 and O6) in the equatorial plane around the Co^II^ ion form a slightly distorted square-planar arrangement, while the slightly distorted octahedral coordination is completed by the two symmetry related O atoms of the water molecules (O5 and O5') in the axial positions. The near equalities of the C1—O1 [1.252 (4) Å], C1—O2 [1.267 (4) Å], C8—O3 [1.251 (4) Å] and C8—O4 [1.260 (4) Å] bonds in the carboxylate groups indicate delocalized bonding arrangement, rather than localized single and double bonds. The average Co—O bond lengths are 2.052 (2) Å (for benzoate oxygens) and 2.128 (2) Å (for water oxygens) (Table 1) close to standard values (Allen *et al.*, 1987). The Co atom is displaced out of the mean-planes of the carboxylate groups (O1/C1/O2) and (O3/C8/O4) by -0.5163 (4) and 0.3155 (4) Å, respectively. The dihedral angles between the planar carboxylate groups (O1/C1/O2) and (O3/C8/O4) and the adjacent benzene rings A (C2—C7) and B (C9—C14) are 51.38 (18) and 39.89 (11) °, respectively. The benzene rings are oriented at a dihedral angle of A/B = 86.09 (10)°.

In the crystal, O-H···O and weak C-H···O hydrogen bonds (Table 2) link the molecules into a three-dimentional supramolecular network. A weak C-H···π interaction is also found in the crystal structure.

### Experimental

The title compound was prepared by the reaction of CoSO_4_.7H_2_O (1.41 g, 5 mmol) in H_2_O (100 ml) with sodium 2-iodobenzoate (2.70 g, 10 mmol) in H_2_O (50 ml) at room temperature. The mixture was filtered and set aside to crystallize at ambient temperature for one week, giving orange single crystals.

### Refinement

Atoms H51, H52, H61 and H62 (for H_2_O) were located in a difference Fourier map and were refined by applying restraints. The C-bound H-atoms were positioned geometrically with C—H = 0.95 Å for aromatic H-atoms, and constrained to ride on their parent atoms, with *U*_iso_(H) = 1.2 × *U*_eq_(C).

### Crystal data

**Table:**

[Co(C_7_H_4_IO_2_)_2_(H_2_O)_2_]	*F*(000) = 1108
*M**_r_* = 588.97	*D*_x_ = 2.277 Mg m^−^^3^
Orthorhombic, *P*2_1_2_1_2_1_	Mo *K*α radiation, λ = 0.71073 Å
Hall symbol: P 2ac 2ab	Cell parameters from 9972 reflections
*a* = 7.5051 (3) Å	θ = 2.7–28.4°
*b* = 10.5639 (4) Å	µ = 4.62 mm^−^^1^
*c* = 21.6723 (9) Å	*T* = 100 K
*V* = 1718.25 (12) Å^3^	Block, orange
*Z* = 4	0.26 × 0.23 × 0.17 mm

### Data collection

**Table:**

Bruker Kappa APEXII CCD area-detector diffractometer	4313 independent reflections
Radiation source: fine-focus sealed tube	4300 reflections with *I* > 2σ(*I*)
Graphite monochromator	*R*_int_ = 0.037
φ and ω scans	θ_max_ = 28.4°, θ_min_ = 1.9°
Absorption correction: multi-scan (*SADABS*; Bruker, 2005)	*h* = −10→10
*T*_min_ = 0.313, *T*_max_ = 0.456	*k* = −13→14
30167 measured reflections	*l* = −29→28

### Refinement

**Table:**

Refinement on *F*^2^	Secondary atom site location: difference Fourier map
Least-squares matrix: full	Hydrogen site location: inferred from neighbouring sites
*R*[*F*^2^ > 2σ(*F*^2^)] = 0.020	H atoms treated by a mixture of independent and constrained refinement
*wR*(*F*^2^) = 0.051	*w* = 1/[σ^2^(*F*_o_^2^) + (0.0209*P*)^2^ + 2.2878*P*] where *P* = (*F*_o_^2^ + 2*F*_c_^2^)/3
*S* = 1.20	(Δ/σ)_max_ = 0.002
4313 reflections	Δρ_max_ = 0.60 e Å^−^^3^
224 parameters	Δρ_min_ = −1.03 e Å^−^^3^
8 restraints	Absolute structure: Flack (1983), 1835 Friedel pairs
Primary atom site location: structure-invariant direct methods	Flack parameter: 0.016 (19)

### Special details

**Table:**

Geometry. All esds (except the esd in the dihedral angle between two l.s. planes) are estimated using the full covariance matrix. The cell esds are taken into account individually in the estimation of esds in distances, angles and torsion angles; correlations between esds in cell parameters are only used when they are defined by crystal symmetry. An approximate (isotropic) treatment of cell esds is used for estimating esds involving l.s. planes.
Refinement. Refinement of F^2^ against ALL reflections. The weighted R-factor wR and goodness of fit S are based on F^2^, conventional R-factors R are based on F, with F set to zero for negative F^2^. The threshold expression of F^2^ > 2sigma(F^2^) is used only for calculating R-factors(gt) etc. and is not relevant to the choice of reflections for refinement. R-factors based on F^2^ are statistically about twice as large as those based on F, and R- factors based on ALL data will be even larger.

### Fractional atomic coordinates and isotropic or equivalent isotropic displacement parameters (Å^2^)

**Table:**

	*x*	*y*	*z*	*U*_iso_*/*U*_eq_	
I1	1.14075 (3)	0.58295 (2)	0.834432 (12)	0.02259 (6)	
I2	0.86998 (3)	−0.04434 (2)	0.858655 (9)	0.01608 (6)	
Co1	0.81243 (5)	0.25155 (4)	0.997594 (19)	0.00670 (8)	
O1	1.0232 (3)	0.2952 (2)	0.86860 (10)	0.0128 (5)	
O2	0.7492 (3)	0.3161 (2)	0.90771 (10)	0.0107 (4)	
O3	0.4106 (3)	0.0744 (2)	0.98386 (11)	0.0136 (5)	
O4	0.7100 (3)	0.0786 (2)	0.98080 (11)	0.0122 (4)	
O5	0.5662 (3)	0.3202 (2)	1.03253 (11)	0.0093 (4)	
H51	0.562 (7)	0.289 (4)	1.0690 (12)	0.030 (14)*	
H52	0.561 (7)	0.4004 (18)	1.037 (2)	0.034 (14)*	
O6	0.8845 (3)	0.1971 (2)	1.08789 (11)	0.0148 (5)	
H61	0.993 (3)	0.190 (5)	1.100 (2)	0.021 (12)*	
H62	0.824 (6)	0.151 (5)	1.113 (2)	0.048 (17)*	
C1	0.8667 (5)	0.3361 (3)	0.86686 (13)	0.0104 (5)	
C2	0.8126 (4)	0.4134 (3)	0.81193 (14)	0.0125 (6)	
C3	0.9090 (5)	0.5170 (3)	0.79051 (15)	0.0161 (7)	
C4	0.8484 (6)	0.5875 (4)	0.74032 (16)	0.0229 (8)	
H4	0.9116	0.6607	0.7273	0.027*	
C5	0.6959 (6)	0.5502 (5)	0.70965 (16)	0.0311 (10)	
H5	0.6560	0.5967	0.6748	0.037*	
C6	0.6007 (6)	0.4452 (5)	0.72940 (19)	0.0329 (10)	
H6	0.4969	0.4192	0.7078	0.040*	
C7	0.6571 (5)	0.3786 (4)	0.78044 (16)	0.0201 (7)	
H7	0.5897	0.3082	0.7945	0.024*	
C8	0.5602 (4)	0.0274 (3)	0.97274 (13)	0.0082 (5)	
C9	0.5600 (4)	−0.1065 (3)	0.94894 (14)	0.0081 (5)	
C10	0.6767 (4)	−0.1532 (3)	0.90444 (13)	0.0088 (6)	
C11	0.6674 (4)	−0.2780 (3)	0.88547 (15)	0.0134 (6)	
H11	0.7435	−0.3072	0.8535	0.016*	
C12	0.5480 (5)	−0.3607 (3)	0.91279 (16)	0.0157 (7)	
H12	0.5450	−0.4471	0.9006	0.019*	
C13	0.4330 (5)	−0.3169 (3)	0.95795 (18)	0.0183 (7)	
H13	0.3521	−0.3733	0.9774	0.022*	
C14	0.4365 (5)	−0.1903 (3)	0.97459 (15)	0.0137 (6)	
H14	0.3531	−0.1599	1.0040	0.016*	

### Atomic displacement parameters (Å^2^)

**Table:**

	*U*^11^	*U*^22^	*U*^33^	*U*^12^	*U*^13^	*U*^23^
I1	0.01863 (11)	0.01494 (11)	0.03419 (13)	−0.00281 (9)	0.00237 (10)	0.00476 (9)
I2	0.01887 (10)	0.01658 (10)	0.01281 (9)	−0.00775 (9)	0.00753 (8)	−0.00351 (8)
Co1	0.00702 (19)	0.00527 (16)	0.00782 (16)	−0.00060 (14)	−0.00006 (14)	−0.00093 (15)
O1	0.0111 (11)	0.0154 (11)	0.0118 (11)	0.0016 (9)	0.0016 (8)	0.0024 (9)
O2	0.0092 (11)	0.0128 (11)	0.0102 (10)	0.0004 (9)	0.0007 (8)	0.0018 (8)
O3	0.0117 (11)	0.0071 (10)	0.0221 (12)	0.0017 (8)	0.0011 (8)	−0.0025 (9)
O4	0.0091 (10)	0.0069 (10)	0.0208 (11)	−0.0004 (8)	0.0004 (9)	−0.0025 (9)
O5	0.0088 (10)	0.0056 (10)	0.0135 (11)	−0.0002 (8)	−0.0002 (8)	−0.0014 (8)
O6	0.0092 (11)	0.0205 (12)	0.0146 (11)	0.0002 (10)	−0.0005 (9)	0.0039 (9)
C1	0.0114 (13)	0.0107 (13)	0.0092 (13)	−0.0034 (12)	0.0005 (12)	−0.0020 (10)
C2	0.0144 (15)	0.0163 (15)	0.0067 (13)	0.0044 (12)	0.0008 (11)	−0.0022 (11)
C3	0.0202 (17)	0.0169 (17)	0.0111 (14)	0.0077 (13)	0.0059 (12)	0.0005 (12)
C4	0.032 (2)	0.0222 (17)	0.0145 (16)	0.0163 (17)	0.0104 (15)	0.0081 (13)
C5	0.039 (2)	0.044 (3)	0.0107 (15)	0.023 (2)	0.0014 (15)	0.0072 (16)
C6	0.028 (2)	0.053 (3)	0.0177 (17)	0.012 (2)	−0.0096 (15)	−0.0045 (19)
C7	0.0167 (17)	0.0296 (19)	0.0139 (15)	0.0014 (15)	−0.0035 (13)	−0.0010 (13)
C8	0.0106 (13)	0.0065 (14)	0.0075 (12)	0.0013 (11)	0.0005 (10)	0.0006 (10)
C9	0.0093 (13)	0.0054 (13)	0.0096 (13)	0.0005 (11)	−0.0006 (11)	−0.0011 (10)
C10	0.0101 (14)	0.0111 (15)	0.0052 (12)	−0.0006 (11)	0.0000 (10)	−0.0003 (10)
C11	0.0139 (16)	0.0139 (15)	0.0124 (14)	0.0004 (12)	0.0018 (12)	−0.0059 (11)
C12	0.0186 (17)	0.0083 (15)	0.0202 (16)	−0.0011 (13)	0.0010 (13)	−0.0066 (12)
C13	0.0198 (17)	0.0116 (16)	0.0235 (17)	−0.0066 (14)	0.0052 (14)	−0.0014 (13)
C14	0.0161 (15)	0.0118 (15)	0.0132 (14)	−0.0038 (13)	0.0063 (12)	−0.0041 (12)

### Geometric parameters (Å, º)

**Table:**

I1—C3	2.102 (4)	C3—C4	1.395 (5)
I2—C10	2.100 (3)	C4—C5	1.381 (7)
Co1—O2	2.118 (2)	C4—H4	0.9500
Co1—O3^i^	2.021 (2)	C5—C6	1.387 (7)
Co1—O4	2.016 (2)	C5—H5	0.9500
Co1—O5	2.124 (2)	C6—C7	1.377 (5)
Co1—O5^i^	2.151 (2)	C6—H6	0.9500
Co1—O6	2.110 (2)	C7—C2	1.400 (5)
O1—C1	1.252 (4)	C7—H7	0.9500
O2—C1	1.267 (4)	C8—C9	1.506 (4)
O3—Co1^ii^	2.021 (2)	C9—C10	1.393 (4)
O3—C8	1.251 (4)	C9—C14	1.397 (4)
O4—C8	1.260 (4)	C11—C10	1.382 (4)
O5—Co1^ii^	2.151 (2)	C11—H11	0.9500
O5—H51	0.855 (19)	C12—C11	1.385 (5)
O5—H52	0.855 (18)	C12—H12	0.9500
O6—H61	0.854 (18)	C13—C12	1.385 (5)
O6—H62	0.86 (2)	C13—H13	0.9500
C2—C1	1.499 (4)	C14—C13	1.385 (4)
C3—C2	1.392 (5)	C14—H14	0.9500
			
O2—Co1—O5	91.32 (9)	C4—C3—I1	116.5 (3)
O2—Co1—O5^i^	91.88 (9)	C5—C4—C3	119.5 (4)
O3^i^—Co1—O6	88.28 (10)	C5—C4—H4	120.2
O3^i^—Co1—O2	88.37 (10)	C3—C4—H4	120.2
O3^i^—Co1—O5	86.32 (9)	C4—C5—C6	120.5 (3)
O3^i^—Co1—O5^i^	93.32 (9)	C4—C5—H5	119.8
O4—Co1—O2	92.32 (9)	C6—C5—H5	119.8
O4—Co1—O3^i^	178.57 (10)	C7—C6—C5	119.8 (4)
O4—Co1—O5	92.41 (9)	C7—C6—H6	120.1
O4—Co1—O5^i^	87.92 (9)	C5—C6—H6	120.1
O4—Co1—O6	91.04 (10)	C6—C7—C2	120.9 (4)
O5—Co1—O5^i^	176.77 (4)	C6—C7—H7	119.5
O6—Co1—O2	176.58 (10)	C2—C7—H7	119.5
O6—Co1—O5	89.15 (9)	O3—C8—O4	127.2 (3)
O6—Co1—O5^i^	87.63 (9)	O3—C8—C9	116.0 (3)
C1—O2—Co1	122.7 (2)	O4—C8—C9	116.8 (3)
C8—O3—Co1^ii^	136.6 (2)	C10—C9—C8	124.7 (3)
C8—O4—Co1	139.0 (2)	C10—C9—C14	117.9 (3)
Co1—O5—Co1^ii^	122.81 (10)	C14—C9—C8	117.3 (3)
Co1—O5—H51	103 (3)	C9—C10—I2	124.6 (2)
Co1^ii^—O5—H51	97 (4)	C11—C10—I2	114.6 (2)
Co1—O5—H52	115 (4)	C11—C10—C9	120.8 (3)
Co1^ii^—O5—H52	110 (4)	C10—C11—C12	120.5 (3)
H51—O5—H52	106 (4)	C10—C11—H11	119.8
Co1—O6—H61	123 (3)	C12—C11—H11	119.8
Co1—O6—H62	127 (4)	C11—C12—H12	120.2
H61—O6—H62	106 (4)	C13—C12—C11	119.6 (3)
O1—C1—O2	125.1 (3)	C13—C12—H12	120.2
O1—C1—C2	117.8 (3)	C12—C13—H13	120.1
O2—C1—C2	117.2 (3)	C14—C13—C12	119.7 (3)
C3—C2—C1	123.5 (3)	C14—C13—H13	120.1
C3—C2—C7	118.5 (3)	C9—C14—H14	119.3
C7—C2—C1	118.0 (3)	C13—C14—C9	121.4 (3)
C2—C3—I1	122.7 (2)	C13—C14—H14	119.3
C2—C3—C4	120.7 (4)		
			
O3^i^—Co1—O2—C1	72.6 (2)	C4—C3—C2—C1	−177.9 (3)
O4—Co1—O2—C1	−108.6 (2)	C4—C3—C2—C7	2.4 (5)
O5—Co1—O2—C1	158.9 (2)	I1—C3—C4—C5	−179.4 (3)
O5^i^—Co1—O2—C1	−20.6 (2)	C2—C3—C4—C5	−3.3 (5)
O2—Co1—O4—C8	−70.4 (3)	C3—C4—C5—C6	1.6 (6)
O5—Co1—O4—C8	21.0 (3)	C4—C5—C6—C7	0.9 (6)
O5^i^—Co1—O4—C8	−162.2 (3)	C5—C6—C7—C2	−1.7 (6)
O6—Co1—O4—C8	110.2 (3)	C6—C7—C2—C1	−179.6 (3)
O2—Co1—O5—Co1^ii^	63.36 (13)	C6—C7—C2—C3	0.1 (5)
O3^i^—Co1—O5—Co1^ii^	151.65 (14)	O3—C8—C9—C10	142.8 (3)
O4—Co1—O5—Co1^ii^	−29.01 (13)	O3—C8—C9—C14	−38.8 (4)
O6—Co1—O5—Co1^ii^	−120.02 (13)	O4—C8—C9—C10	−39.0 (4)
Co1—O2—C1—O1	16.8 (4)	O4—C8—C9—C14	139.4 (3)
Co1—O2—C1—C2	−164.0 (2)	C8—C9—C10—I2	−2.7 (4)
Co1^ii^—O3—C8—O4	14.0 (5)	C8—C9—C10—C11	179.7 (3)
Co1^ii^—O3—C8—C9	−168.0 (2)	C14—C9—C10—I2	178.9 (2)
Co1—O4—C8—O3	−13.8 (5)	C14—C9—C10—C11	1.4 (5)
Co1—O4—C8—C9	168.2 (2)	C8—C9—C14—C13	−176.5 (3)
C3—C2—C1—O1	−51.1 (4)	C10—C9—C14—C13	2.0 (5)
C3—C2—C1—O2	129.7 (3)	C12—C11—C10—I2	178.7 (3)
C7—C2—C1—O1	128.6 (3)	C12—C11—C10—C9	−3.5 (5)
C7—C2—C1—O2	−50.7 (4)	C13—C12—C11—C10	2.2 (5)
I1—C3—C2—C1	−2.0 (4)	C14—C13—C12—C11	1.1 (6)
I1—C3—C2—C7	178.3 (2)	C9—C14—C13—C12	−3.2 (6)

Symmetry codes: (i) *x*+1/2, −*y*+1/2, −*z*+2; (ii) *x*−1/2, −*y*+1/2, −*z*+2.

### Hydrogen-bond geometry (Å, º)

**Table:**

*D*—H···*A*	*D*—H	H···*A*	*D*···*A*	*D*—H···*A*
O5—H51···O1^ii^	0.86 (3)	1.64 (3)	2.486 (3)	167 (5)
O6—H61···O2^i^	0.86 (2)	1.92 (2)	2.742 (3)	161 (4)
C4—H4···O1^iii^	0.95	2.56	3.364 (4)	142
C13—H13···O4^iv^	0.95	2.58	3.495 (4)	162

Symmetry codes: (i) *x*+1/2, −*y*+1/2, −*z*+2; (ii) *x*−1/2, −*y*+1/2, −*z*+2; (iii) −*x*+2, *y*+1/2, −*z*+3/2; (iv) *x*−1/2, −*y*−1/2, −*z*+2.
